# Reflections of digital slavery in physical inactivity: examining gender-based physical activity attitudes and smartphone addiction among university students

**DOI:** 10.3389/fpubh.2025.1742639

**Published:** 2026-01-05

**Authors:** Unsal Altinisik, Hasan Guler, Ozkan Isik, Laurentiu-Gabriel Talaghir, Liliana Nanu, Paula Ivan

**Affiliations:** 1 Faculty of Sport Sciences, Aydin Adnan Menderes University, Aydin, Türkiye; 2 Faculty of Sport Sciences, Balikesir University, Balikesir, Türkiye; 3 Sports Sciences Application and Research Center, Balikesir University, Balikesir, Türkiye; 4 Faculty of Physical Education and Sport, Dunarea de Jos University of Galati, Galati, Romania

**Keywords:** digital addiction, gender gap, physical activity, smartphone addiction, social habits

## Abstract

**Purpose:**

The purpose of this study was to examine the gender-based physical activity attitudes of university students according to their smartphone addiction levels.

**Methods:**

The study was designed using a relational screening model. One thousand twenty-one university students participated in the study voluntarily and data were collected using an online survey method. In addition to the demographic characteristics of the participants, the Smartphone Addiction Scale–Short Form and the Cognitive Behavioral Physical Activity Scale were used in the study. Data were analyzed using the SPSS package program. Hierarchical and non-hierarchical cluster analyses were applied to determine the participants’ smartphone addiction levels, and differences in physical activity attitudes according to gender and smartphone addiction level were tested with Univariate Analysis of Variance.

**Results:**

A significant difference was found in participants’ physical activity attitudes according to their smartphone addiction levels (*p* = 0.001). Individuals with low smartphone addiction had higher physical activity attitude scores than those with moderate and high addiction levels. Regarding gender, men had significantly higher physical activity attitude scores than women (*p* = 0.001). However, the interaction between gender and smartphone addiction was found to have no significant effect on physical activity attitudes (*p* = 0.053).

**Conclusion:**

It was observed that attitudes toward physical activity decreased as smartphone addiction levels increased. This suggests that smartphone addiction negatively impacts young people’s active lifestyle habits and increases their tendency toward sedentary behavior. By demonstrating the effects of digital addiction on attitudes toward physical activity, the study provides important findings regarding the sustainability of young people’s healthy lifestyle habits.

## 1 Introduction

Internet usage in Türkiye has reached 90.9%, and this rate is particularly high among young people (1). Young people spend several hours a day on mobile devices for communication, education, and entertainment (2). While smartphones facilitate access to information and social interaction, excessive use has been associated with distraction, sleep disturbances, and physical inactivity (3–5). The increase in digital addiction, especially among university students, reduces interest and participation in physical activity and contributes to the spread of a sedentary lifestyle among young people (6). Physical activity is one of the main determinants of both physical health and psychological well-being (7, 8), yet increased smartphone use has been shown to weaken active lifestyle habits and attitudes toward physical activity (9). In this context, examining the relationship between smartphone addiction and attitudes toward physical activity in young adults is important for the sustainability of healthy daily lifestyle behaviors in the digital age.

The increase in smartphone usage among young people shapes daily life habits and motivational processes related to physical activity (10–12). Smartphone use also varies by gender, with men generally using digital devices more intensively for gaming and competitive activities, while women tend to use them more for social interaction and communication (13–15). At the same time, physical activity behavior is influenced by gendered expectations, perceived competence, and opportunities for participation (14, 16). Men often prefer competitive and performance-oriented activities, whereas women tend to prioritize social support and enjoyment (15). Because social approval and relational commitment are more dominant in women, and competition and performance orientation are more dominant in men, these differences may influence their attitudes toward physical activity. Attitudes toward physical activity are a key determinant of maintaining active lifestyle behaviors (17, 18). However, smartphone use can negatively affect this process by weakening time management, self-control, and physical awareness skills and by reinforcing sedentary behaviors and negative attitudes toward physical activity (19–22). All of these behavioral patterns can be conceptually linked to the Components Model of Addiction and Self-Determination Theory. Despite this, no research has been found that directly examines the effects of smartphone addiction on attitudes toward physical activity. Furthermore, the lack of evidence assessing this relationship within the framework of gender differences represents a significant gap in the field.

The Components Model (23), developed within the framework of behavioral addiction, states that a behavior is considered addictive when it has six core characteristics: salience, mood modification, tolerance, withdrawal, conflict, and relapse. In this model, the addictive behavior becomes central in an individual’s life and a dominant activity that directs cognitive processes, emotions, and actions. Individuals may engage in this behavior to avoid negative emotions or to obtain relief, and over time the frequency or duration of the behavior increases to achieve the same effect. When the behavior is discontinued, anxiety, tension, and withdrawal symptoms may arise, leading to conflicts in social relationships and in academic or professional life and often resulting in relapse despite attempts to quit (23–25). Thus, the Components Model provides a conceptual framework for understanding the development and maintenance of addictive behaviors and offers a theoretical basis for examining the impact of smartphone addiction on attitudes and behaviors toward physical activity.

Self-Determination Theory argues that individuals conduct their behaviors based on intrinsic motivation, autonomy, and feelings of competence (26). According to this theory, sustained engagement in health-related behaviors such as physical activity depends on the satisfaction of basic psychological needs for autonomy, competence, and relatedness (27). Smartphone addiction can weaken intrinsic motivation for physical activity by limiting autonomous behavior, reducing face-to-face social interaction, and restricting opportunities to experience competence in physical activity contexts. As young people’s digital addiction levels increase, their desire to participate in physical activity and their positive attitudes toward such activities may decrease (26, 28). In this sense, while the Components Model suggests that smartphone addiction may lead to withdrawal from physical activity, Self-Determination Theory helps to explain how this process is reinforced through the loss of autonomy and motivation.

The placement of smartphones at the center of life has led to radical changes in behavioral and physical habits in recent years (29). Intensive phone use, especially in young adulthood, increases sedentary lifestyles by affecting many areas from social relationships to daily time management (19, 20, 22). Although there are many studies in the literature on the effects of smartphone addiction on academic success, sleep quality, and psychological well-being (30–34), these studies have mainly focused on these outcomes rather than attitudes toward physical activity. In this context, the present study aimed to examine university students’ attitudes toward physical activity in relation to their smartphone addiction levels and gender.

H_1_: There is a significant difference in the attitudes of university students towards physical activity according to their smartphone addiction levels.

H_2_: There is a significant difference in the attitudes of university students towards physical activity according to their gender.

H_3_: The interaction between smartphone addiction level and gender has a significant effect on attitudes toward physical activity.

## 2 Methods

### 2.1 Research model

This study employed a cross-sectional, quantitative research design. A general survey model was used to reach general conclusions through data obtained from a representative sample within a population consisting of numerous elements (35). In the study, a relational screening model was used to examine the attitudes of university students toward physical activity according to their smartphone addiction levels.

### 2.2 Study sample

Students studying at state universities in the Marmara Region of Türkiye were included in the study. A convenience sampling method was employed, as the target population consisted of university students who could be efficiently reached through institutional communication channels. To be eligible for participation, students were required to be enrolled as undergraduate students at a state university in the Marmara Region, to be at least 18 years of age, to use a smartphone, and to voluntarily complete the online questionnaire. Students who reported not using a smartphone, who were younger than 18 years, or who submitted questionnaires with missing or inconsistent responses were excluded from the analyses. Convenience sampling is frequently used in educational and behavioral research when researchers aim to collect data from an accessible population within practical time and resource constraints (36). This method allowed researchers to collect data quickly from easily accessible individuals. However, it was acknowledged that the generalizability of the findings may be limited due to the non-probability sampling method. To mitigate this limitation, students from different faculties and departments in different provinces of the Marmara Region (Istanbul, Tekirdağ, Edirne, Kırklareli, Balıkesir, Çanakkale, Bursa, Bilecik, Sakarya, Kocaeli, and Yalova) were included in the study. According to Sekaran (37), a sample size of 384 is considered sufficient for research when the population size exceeds 10,000,000. However, in this study, a larger sample size of 1.021 participants was used to increase statistical power and support the reliability of the results.

### 2.3 Data collection tools

All participants were included in the study voluntarily, were informed in advance about the purpose of the study, and their consent was obtained following ethical principles. To collect data in the study, the Personal Information Form prepared by the researcher to determine the short demographic characteristics of the participants, as well as the Cognitive Behavioral Physical Activity Scale and the Smartphone Addiction Scale-Short Form (SAS-SF), were used. Data were collected via an online survey method, and each participant responded to the questions for an average of 5–7 min.

#### 2.3.1 Personal information form

Developed by the researchers, the form includes questions to determine the demographic characteristics of the participants (age, gender, GPA, daily smartphone usage). This form was developed by the researchers and has been used in previous studies conducted with similar university student samples (17, 38). A total of 1,021 university students participated in the study voluntarily. The research team reviewed all answers to ensure data integrity and excluded incomplete or inconsistent responses from the statistical analysis. The mean age of the participants was 21.6 ± 2.3 years, and 53.5% were female (*n* = 546) and 46.5% were male (*n* = 475). When the participants’ GPAs were examined, it was found that 17.8% were ≤2.00, 25.1% were <2.00–2.51, 33.4% were >2.50–3.00, 20.1% were <3.00–3.50, and 3.6% were <3.50–4.00. When the daily smartphone usage time of the participants was examined, it was determined that 6.2% of the students used smartphones for less than 1 h, 29.7% for 1–2 h, 39.5% for 3–4 h, and 24.7% for 5 h and above (Table 1).

**Table 1:** Demographic characteristics of participants.

Age	Mean	S. D.
	21.6	2.3
Gender	f	%
Female	546	53.5
Male	475	46.5
GPAs	f	%
≤2.00	182	17.8
>2.00–2.50	256	25.1
>2.50–3.00	341	33.4
>3.00–3.50	205	20.1
>3.51–4.00	37	3.6
Daily smartphone usage	f	%
Less than 1 h	63	6.2
1–2 h	303	29.7
3–4 h	403	39.5
5 h and above	252	24.7
N: 1021		

#### 2.3.2 Smartphone addiction scale-short form

The SAS-SF, developed by Kwon et al. (53) and adapted to the Turkish language by Noyan et al. (54), has a 10-item, single-factor structure. The scale was rated on a 6-point Likert-type scale (Strongly disagree = 1, Disagree = 2, Partially disagree = 3, Partially agree = 4, Agree = 5, Strongly agree = 6) and has no reverse items. The lowest possible score was 10, and the highest was 60. A higher score indicates a higher risk of addiction. Cronbach’s Alpha internal consistency coefficient was 0.87, and the test–retest reliability coefficient was 0.93. This result demonstrates that the scale has a high level of internal consistency and reliability (39).

#### 2.3.3 The cognitive behavioral physical activity scale

The Cognitive Behavioral Physical Activity Scale, developed by Schembre et al. (55) and adapted into Turkish by Eskiler et al. (56), has a 15-item structure with 3 factors (outcome expectation, self-regulation, personal barriers). The scale was rated on a 5-point Likert-type scale (Strongly disagree = 1, Disagree = 2, Neither agree nor disagree = 3, Agree = 4, Strongly agree = 5), and the items in the personal barriers dimension were negative. The scale’s internal consistency coefficient was 0.84. Outcome expectation was 0.85, self-regulation was 0.79, and personal barriers were 0.64. These findings demonstrate that the scale has adequate internal consistency (39).

### 2.4 Ethical approval

This study was ethically approved by the Balıkesir University Health Sciences Non-invasive Research Ethics Committee, with decision number 2025/333. No personally identifying information (such as name, student ID number, e-mail address, or IP address) was collected in the online questionnaire. All responses were recorded anonymously and stored in an encrypted file on a password-protected computer, and only the research team had access to the dataset. Participation was entirely voluntary, and students could withdraw from the survey at any point without providing a reason. Because the survey was administered online and relied on self-selection, non-response bias cannot be ruled out; students with different levels of interest in physical activity or different patterns of smartphone use may have been differentially likely to participate. This issue was taken into account when interpreting the findings and is acknowledged as a limitation of the study.

### 2.5 Statistical analysis

Data were analyzed using the IBM SPSS package program (IBM Corp., Armonk, NY, United States). Mean and standard deviation were used as descriptive statistics. Data normality was checked by ensuring that skewness and kurtosis values were within ±2 (40). In addition, Pearson correlation coefficients were calculated to examine the bivariate relationship between smartphone addiction (SAS-SF scores) and attitudes toward physical activity. To classify participants according to their smartphone addiction levels, a two-step cluster analysis combining hierarchical and non-hierarchical methods was employed (41). First, a hierarchical cluster analysis using Ward’s method was conducted to explore the appropriate number of clusters and to obtain initial cluster centers (42, 43). Inspection of the agglomeration schedule and the interpretability of the solutions indicated that a three-cluster solution was most appropriate. Second, a non-hierarchical k-means cluster analysis was performed with the number of clusters fixed at three in order to refine cluster membership. Cluster validity was examined by inspecting the distances between the final cluster centers and by testing between-cluster differences in SAS-SF scores using ANOVA. In the absence of widely accepted cut-off scores for the SAS-SF in Turkish university students, this empirical, cluster-based approach was preferred over the use of arbitrary thresholds to define addiction levels. Based on their mean SAS-SF scores within the theoretical range of the scale, the three clusters were labeled as low, medium, and high smartphone addiction. Additionally, a univariate analysis of variance (ANOVA) was used to determine the participants’ physical activity attitudes according to their gender and smartphone addiction levels and to determine the effect of the interaction between smartphone addiction and gender on physical activity attitudes. When significant main effects were detected, post-hoc pairwise comparisons with Bonferroni adjustment were conducted to examine specific differences between the smartphone addiction clusters.

## 3 Results

The distribution and means of the clusters regarding smartphone addiction results were given in Table 2.

**Table 2:** Final cluster center.

Variables	Cluster 1	Cluster 2	Cluster 3	Mean
Smartphone addiction	1.90	3.40	4.85	3.13
6-point Likert type				

When Table 3 was examined, it was determined that there were 446 (43.7%) participants in the first (low) cluster, 202 (19.8%) in the second (medium) cluster, and 373 (36.5%) in the third (high) cluster. The distances between cluster centers ranged from 1.451 to 2.959. The highest distance was observed between the low and high clusters (2.959), and the lowest distance was observed between the medium and high clusters (1.451). This result indicates a significant and distinct separation between the clusters. In addition, a one-way analysis of variance revealed that the difference between the groups was significant at the 0.01 level. Therefore, cluster analysis resulted in three distinct groups of participants with smartphone addiction levels: low, medium, and high.

**Table 3:** Inter-cluster distance and cluster size.

Cluster	N	Cluster size (%)	Inter-cluster distance		
			Cluster 1	Cluster 2	Cluster 3
Low	446	43.7		1.508	2.959
Medium	202	19.8	1.508		1.451
High	373	36.5	2.959	1.451	
Total	1,021	100			

**Figure 1:**
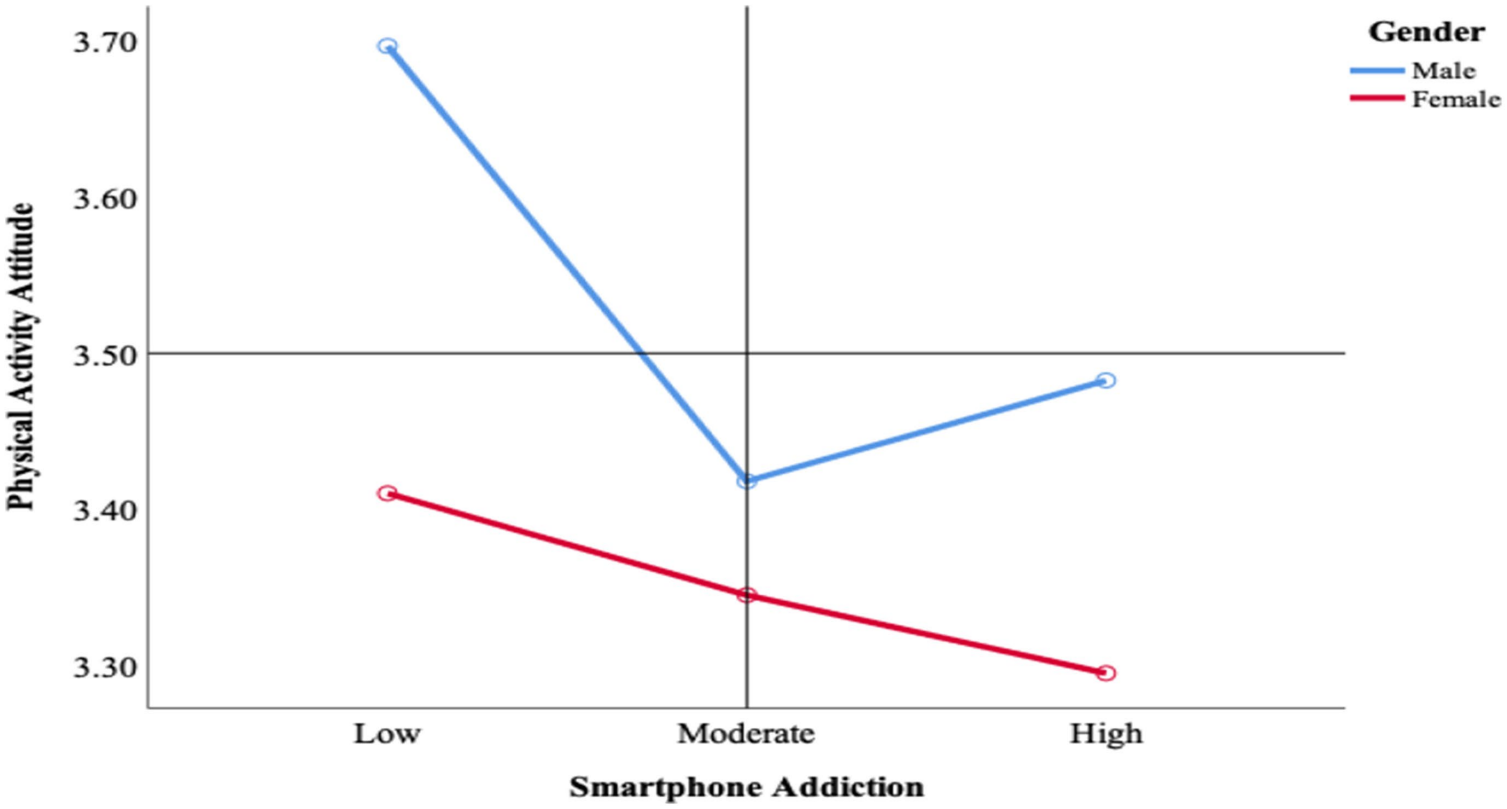
Average scores for physical activity attitude of male and female with different levels of smartphone addiction.

When the analysis results were examined, it was observed that participants’ physical activity attitudes differed by gender (*p* = 0.001). Additionally, a significant difference was found in participants’ physical activity attitudes according to their smartphone addiction levels (*p* = 0.001). However, the corresponding partial eta squared values indicated small effect sizes, suggesting that the practical differences between groups were modest despite being statistically significant. Post-hoc pairwise comparisons with Bonferroni adjustment showed that the high smartphone addiction group had significantly higher physical activity attitude scores than both the low and medium addiction groups, whereas no significant difference was observed between the low and medium groups. However, when the interaction between smartphone addiction and gender was examined, no significant difference in physical activity attitudes was observed (*p* = 0.053; Table 4). The average scores for physical activity attitudes of men and women at different smartphone addiction levels were presented in Figure 1. Visual inspection of these means indicated that, among male students, physical activity attitudes were slightly higher in the high-addiction cluster than in the medium-addiction cluster.

**Table 4:** Results of the difference analysis on physical activity attitude on participants’ smartphone addiction levels and gender.

Source	Sum of squares	df	Mean square	F	*p*	η^2^
Corrected model	12.478	5	2.496	9.287	0.001	0.044
Intercept	10705.641	1	10705.641	39838.208	0.001	0.975
Gender	7.533	1	7.533	28.032	0.001	0.027
Smartphone addiction levels	4.547	2	2.274	8.461	0.001	0.016
Gender * smartphone addiction levels	1.582	2	0.791	2.944	0.053	0.006
Error	272.759	1,015	0.269			
Total	12177.018	1,021				
Corrected total	285.237	1,020				

Figure 1 shows the distribution of physical activity attitude averages for men and women according to their smartphone addiction levels. Male participants’ attitudes toward physical activity were highest at low smartphone addiction levels, while physical activity attitude scores decreased to the lowest level at moderate smartphone addiction levels and slightly increased at high smartphone addiction levels. Female participants generally had lower physical activity attitude scores than male participants. Additionally, as women’s smartphone addiction levels increased, a gradual decrease in their physical activity attitude scores occurred.

## 4 Discussion

This study aimed to examine university students’ attitudes toward physical activity according to their smartphone addiction levels. According to the analysis results, a significant difference was found in the participants’ physical activity attitudes according to their smartphone addiction levels. This finding supports the H_1_ hypothesis of our research. As participants’ smartphone addiction levels increased, their attitudes toward physical activity decreased. These findings suggest that smartphone addiction negatively impacts individuals’ active lifestyles and increases their tendency toward a sedentary lifestyle. Participants with low smartphone addiction had higher attitudes toward physical activity. This can be interpreted as increasing the use of technology may reduce the desire to participate in physical activities.

Many studies support the findings of our research. In a study conducted on Chinese university students, Gong et al. (44) reported a negative relationship between physical activity level and smartphone addiction. Aligül and Tolukan (57) found a negative relationship between smartphone addiction and motivation to participate in physical activity in sports sciences students. In other words, they reported that as smartphone addiction increases, motivation for physical activity decreases. Kim and Lee (45), in a study on adolescents, reported that adolescents who perform in more physical activity per week have a lower risk of smartphone addiction, and that regular physical activity significantly reduces the risk of smartphone addiction. Lin et al. (46) similarly found that physical activity levels decrease as smartphone addiction increases in university students. Furthermore, Buke et al. (47) examined the impact of smartphone addiction on physical activity levels in sports science students and found a negative relationship between smartphone addiction and physical activity levels in physical education and sports teaching and sports management students. These studies, consistent with the findings of the current study, highlight that increased smartphone addiction has a negative impact on individuals’ participation in physical activity and their positive attitudes toward sports.

The three-cluster solution obtained in this study also points to meaningful behavioral profiles of smartphone use. Students in the low-addiction cluster seem to use their phones mainly for functional purposes such as communication, information seeking, and academic tasks, with relatively limited signs of loss of control or conflict. Those in the medium-addiction cluster appear to combine this instrumental use with more frequent checking and entertainment-oriented activities, reflecting an intermediate level of salience and preoccupation. In contrast, the high-addiction cluster reflects more intensive and persistent use patterns that are consistent with the Components Model of Addiction, including stronger preoccupation with the device, greater difficulty in limiting use, and a tendency to integrate smartphone use into most daily routines. These behavioral distinctions, in line with this model (23, 25), help to contextualize the differences in physical activity attitudes observed across addiction levels.

Findings regarding the gender variable indicate that male participants’ attitude scores toward physical activity were significantly higher than those of female participants. This result supports hypothesis H_2_ of our research. Our research was similar to the results of previous studies (16, 38, 58). Men’s higher physical activity scores may be due to their greater involvement in activities, their perception of themselves as more athletically competent, and their greater access to physical activities. For women, gender roles may limit participation in physical activity. In the Turkish context, traditional gender norms, family expectations and safety concerns may restrict young women’s opportunities to engage in regular physical activity, whereas young men are more often encouraged to participate in competitive and performance-oriented sports and have easier access to male-dominated sport environments. These gendered socialization processes and differences in perceived competence and social support are consistent with previous research and may contribute to more positive attitudes toward physical activity among men compared with women. In addition, women’s more intensive use of phones for social communication and entertainment purposes (48) may negatively affect their attitudes by reducing the time spent on physical activity. These results suggest that attitudes toward physical activity are shaped by the combined influence of social and behavioral factors, and in particular, gender-based social expectations and technology use habits may be decisive in shaping their physical activity attitudes.

Another finding in our study was that the interaction between gender and smartphone addiction was not significant. This suggests that smartphone addiction affects physical activity attitudes similarly in men and women. In other words, as addiction levels increase, positive attitudes toward physical activity decrease in both gender groups. When the literature was examined, Numanoğlu-Akbaş et al. (49) stated in their study with university students that there was no significant difference between male and female students in terms of smartphone addiction level. They also found no significant gender difference between physical activity levels and addiction. Wu and Chou (50) and Song et al. (51) similarly found that gender did not have a significant effect on variables associated with smartphone addiction. A study conducted in India found a strong negative correlation between smartphone addiction and physical activity. However, no significant gender-based difference was observed (52). Thus, it can be argued that the impact of smartphone addiction on physical activity attitudes is similar, regardless of gender.

The Components Model suggests that individuals experience functional impairments in other areas of their lives due to excessive focus on a specific behavior (23). According to this model, smartphone addiction manifests itself through symptoms such as salience, mood regulation, tolerance, and conflict (25). In other words, smartphone addiction negatively impacts individuals’ time management, leading them to abandon healthy lifestyles such as physical activity. Consequently, as smartphone addiction increases, individuals’ motivation to participate in physical activity decreases and sedentary behaviors increase (52). On the other hand, Self-Determination Theory emphasizes that intrinsic motivation can be sustained by the satisfaction of the basic psychological needs of autonomy, competence, and relatedness that guide individuals’ behavior (26). Smartphone addiction prevents an individual from satisfying these needs, weakening their capacity for autonomous behavior (28). This can lead to a decrease in intrinsic motivation for physical activity and a withdrawal from an active lifestyle (27). In this context, it can be argued that while the Components Model suggests that smartphone addiction leads to withdrawal from physical activity, Self-Determination Theory reinforces this process through a loss of autonomy and motivation.

## 5 Conclusion

As a result, it was determined that university students’ attitudes toward physical activity decreased as smartphone addiction levels increased. Male students had higher attitude scores than female students, and this effect did not differ by gender. We recommend that researchers examine the impact of digital addiction on physical activity across different age groups, branches, and psychological variables. We also recommend that practitioners design programs that balance students’ smartphone use, encourage physical activity, and increase awareness of healthy lifestyles.

### 5.1 Limitations and strengths of the study

This study has several limitations that should be considered. First, the sample was obtained through convenience sampling from state universities in the Marmara Region of Türkiye. This non-probability sampling strategy may limit the generalizability of the findings to other regions, age groups, or non-student populations and may introduce sampling and non-response bias. Second, all variables were measured using self-report questionnaires, which are susceptible to recall errors and social desirability tendencies, particularly in relation to physical activity and smartphone use. Third, the cross-sectional design does not allow for causal inferences about the direction of the relationships between smartphone addiction and attitudes toward physical activity. In addition, smartphone addiction levels were defined empirically through a three-cluster solution rather than established clinical cut-off scores, and the effect sizes observed in the group comparisons were small, indicating that the group differences identified are statistically reliable but modest in magnitude.

Despite these limitations, the study has several noteworthy strengths. The sample size (*n* = 1,021) is relatively large for this type of research and provides adequate statistical power for the analyses conducted. Validated and widely used instruments were employed to assess smartphone addiction (SAS-SF) and attitudes toward physical activity, which supports the reliability and construct validity of the measurements. The use of a two-step cluster analysis in combination with Univariate ANOVA allowed for a nuanced examination of how empirically derived smartphone addiction profiles relate to physical activity attitudes, while also considering gender and its interaction with addiction levels. Furthermore, the study is grounded in established theoretical frameworks, namely the Components Model of Addiction and Self-Determination Theory, and addresses an understudied relationship in the literature by linking digital behavior patterns with attitudes toward physical activity in a large sample of university students.

### 5.2 Recommendations for future studies and practitioners

For future research, it would be valuable to replicate and extend the present findings with more diverse and representative samples, including students from different regions, private universities and non-student young adults. Longitudinal and experimental designs are needed to clarify the causal links between smartphone addiction and attitudes toward physical activity, for example by examining whether reductions in problematic smartphone use lead to improvements in physical activity attitudes and behavior over time. Future studies could also compare cluster-based classifications with different operationalizations of smartphone addiction (e.g., clinically oriented cut-off scores, latent class analyses) and investigate whether similar patterns emerge across methods. In addition, incorporating objective indicators such as accelerometer-based physical activity data or smartphone usage logs alongside self-report measures would help reduce common method bias and provide a more comprehensive picture of the relationship between digital behaviors and active lifestyle patterns. Finally, qualitative studies exploring students’ subjective experiences of balancing smartphone use and physical activity, as well as perceived barriers and facilitators, may deepen the understanding of the mechanisms underlying the quantitative associations observed in this study.

For practitioners and policymakers in higher education and public health, the findings underscore the importance of jointly addressing digital well-being and promoting physical activity. Universities could develop integrated programs that encourage students to take active breaks from screen-based activities, incorporate movement into daily routines and participate in campus recreation opportunities. Interventions may include psychoeducational workshops on managing smartphone use, digital detox challenges linked with physical activity events and the use of mobile applications that support goal-setting and self-monitoring of both screen time and physical activity. Given the gender differences observed in physical activity attitudes, it is also important to design gender-sensitive strategies that consider cultural and social factors, such as safety, social support and access to facilities, particularly for female students. Collaborations between student affairs, counseling centers, faculties of sport sciences, and information technology departments may facilitate the implementation of multidimensional programs that promote a balanced use of smartphones and foster sustainable active lifestyles among university students.

## Data Availability

The raw data supporting the conclusions of this article will be made available by the authors, without undue reservation.
